# Impact of national volume-based procurement on physicians’ antimicrobial prescribing behaviours: an interrupted time series analysis of 1200 prescriptions in a tertiary hospital

**DOI:** 10.1080/07853890.2026.2624209

**Published:** 2026-02-08

**Authors:** Zhenzhen Du, Jiapei Yao, Xinru Liu, Jinhong Gong, Di Yang, Xin Li, Dan Su, Xindie Zhou, Jingjing Shang

**Affiliations:** a School of Pharmacy, Dalian Medical University College of Pharmacy, Dalian, China; b Department of Pharmacy, The Second People’s Hospital of Changzhou, The Third Affiliated Hospital of Nanjing Medical University, Changzhou Medical Center, Changzhou, China; c Department of Orthopedics, The Second People’s Hospital of Changzhou, The Third Affiliated Hospital of Nanjing Medical University, Changzhou Medical Center, Changzhou, China; d Department of Pharmaceutical Regulatory Science and Pharmacoeconomics, School of Pharmacy, Nanjing Medical University, Nanjing, China

**Keywords:** Impact of national volume-based procurement, antimicrobial prescribing behaviour, interrupted time series analysis, drug expenditure, antimicrobial use structure, special-grade antimicrobial agents

## Abstract

**Objective:**

This study aimed to investigate the impact of the National Volume-Based Procurement (NVBP) policy on antimicrobial use intensity, prescribing structure, and medical costs among inpatients in a tertiary hospital in China.

**Methods:**

This study retrospectively extracted 1200 inpatient prescriptions from a tertiary hospital between April 2019 and March 2024. Using interrupted time series (ITS) analysis, the study assessed the immediate-level changes and long-term trend effects of the NVBP policy on medication indicators such as defined daily doses (DDDs) and costs.

**Results:**

Following NVBP implementation, antimicrobial prescribing structure changed significantly, with β-lactam utilization decreasing by 13.85%. The combination rate of β-lactams + fluoroquinolones fell significantly by 75.93%, while overall combination therapy remained unchanged. Use of special-grade antimicrobials showed a numerical increase (+31.98%), although not statistically significant. Cost indicators decreased significantly following policy implementation. ITS analysis further revealed a significant immediate decrease in the proportion of DDDs for NVBP antimicrobial agents (*β_2_* = −15.464 units, *p* = 0.009), followed by a sustained upward trend (*β_3_* = 1.400/month, *p* < 0.001). Similarly, DDDs of NVBP antimicrobial agents showed a significant upward trend (*β_3_* = 0.135 units/month, *p* = 0.017) after the policy.

**Conclusion:**

This study demonstrates that while the NVBP policy significantly reduced patient drug expenditures and promoted the use of NVBP varieties and nonrestricted antimicrobial agents, it also increased the proportion of special-grade antimicrobials agents, suggesting the need for caution against potential antimicrobial resistance and strengthened supervision of prescription.

## Key messages

1. The National Volume-Based Procurement policy significantly reduced antimicrobial drug expenditures and patient out-of-pocket costs in a Chinese tertiary hospital.
2. The policy was associated with increased use of special-grade antimicrobials, highlighting potential risks for antimicrobial resistance.
3. Future drug procurement policies should integrate with antimicrobial stewardship programs to balance cost control and rational antibiotic use.

## 1. Introduction

China’s large population base places considerable pressure on the country’s healthcare system, resulting in the long-standing problem of high medical costs, driven largely by excessively high drug prices [1]. To alleviate patients’ financial burden and address the problem of excessively high drug prices, the Chinese government launched a pilot of the National Volume-Based Procurement (NVBP) policy in 2018. At its core, NVBP organizes public healthcare institutions into purchasing alliances to collectively procure selected drugs. In exchange for guaranteed, substantial purchase volumes, pharmaceutical manufacturers offer significant price discounts. The primary goals of this policy are to directly lower drug prices, thereby reducing the financial burden on both patients and the national health insurance fund. For participating hospitals, the policy includes specific performance assessments related to fulfilling the agreed procurement volumes. By lowering drug prices and promoting the prioritization and use of selected drugs as well as generic substitution, the policy has achieved significant cost savings [2,3]. For example, after the ‘4 + 7’ pilot, the utilization rate of selected drugs increased by 52%, with the rate of generic substitution reaching 17.08% [4,5]. Specific data show that the prices of selected antibacterial drugs dropped sharply, their sales volume grew by 36.60%, and the estimated cost savings amounted to 1.9378 million yuan [6,7].

Regarding antibacterial drugs, the utilization rate of selected antibacterial drugs in one tertiary hospital averaged 85.31%, with some reaching 100% [8]. However, unintended prescribing changes may also occur, such as downgrading to lower-level antibiotics due to efficacy concerns or new price advantages, or an increased preference for higher-level broad-spectrum drugs [8,9]. Some studies have reported a significant increase in the usage volume of related antibiotics post-NVBP policy implementation, indicating the need for vigilance against the risk of antibiotic overuse [2]. Antimicrobial resistance (AMR) poses a pressing global health threat. In 2019, it was linked to approximately 5 million deaths worldwide, with projections suggesting a rise to 10 million annually by 2050 [10,11]. In financial terms, AMR causes global economic losses of approximately 100 trillion US dollars annually [12]. In China, antimicrobial consumption accounts for roughly 10% of the global total, with usage rates among the highest internationally [13]. Surveillance data also indicate a concerning rise in resistance rates among clinically isolated bacteria, particularly carbapenem-resistant Gram-negative bacilli [9,14]. The medical burden and public health risks associated with AMR have attracted considerable attention from the international community, with the 2024 United Nations General Assembly calling for the creation of a global collaborative governance framework to address this issue [15].

At present, several studies on antimicrobial drug management focus on macro-level changes in total drug sales, fluctuations in medical insurance expenditures, or normative analysis of policy texts [8]. However, these perspectives cannot provide deep insights into the actual implementation effects of policies in clinical practice and subtle changes in the prescribing behaviours of physicians. This study aims to fill this critical gap by focusing on micro-level prescribing practices, specifically, the real-world antibiotic prescribing patterns derived from inpatient prescription data within a single tertiary institution.

## 2. Materials and methods

### 2.1. Study design and setting

This study adopted a retrospective observational design to quantitatively assess the impact of the NVBP policy on physicians’ antimicrobial prescribing behaviours. The study was conducted in a large tertiary grade A general hospital located in Eastern China, which has approximately 2,534 inpatient beds and handles about 2.48 million outpatient visits and 125,900 inpatient discharges annually (2024 data). Its patient composition and disease spectrum are representative of peer tertiary hospitals in China. Prescription data collected at multiple time points pre- and post-NVBP policy implementation were used for analysis. The official implementation date of the NVBP policy in the hospital (January 1, 2022, the effective date of the fifth national procurement round results) was used as the clear key intervention point. The study period was approximately 60 months, spanning from 33 months before to 27 months after NVBP policy implementation, to effectively distinguish the immediate effects of the policy and ensure adequate capture of long-term trend changes. This study was approved by the Ethics Committee of The Second People’s Hospital of Changzhou, The Third Affiliated Hospital of Nanjing Medical University (Approval No.: [2025] KY212-01) and was conducted in accordance with the principles of the Declaration of Helsinki. This study was registered at the Chinese Clinical Trial Registry (Registration No.: ChiCTR2500111392). Informed consent was waived owing to the retrospective nature of the study.

### 2.2. Data source and collection

**Figure 1.**
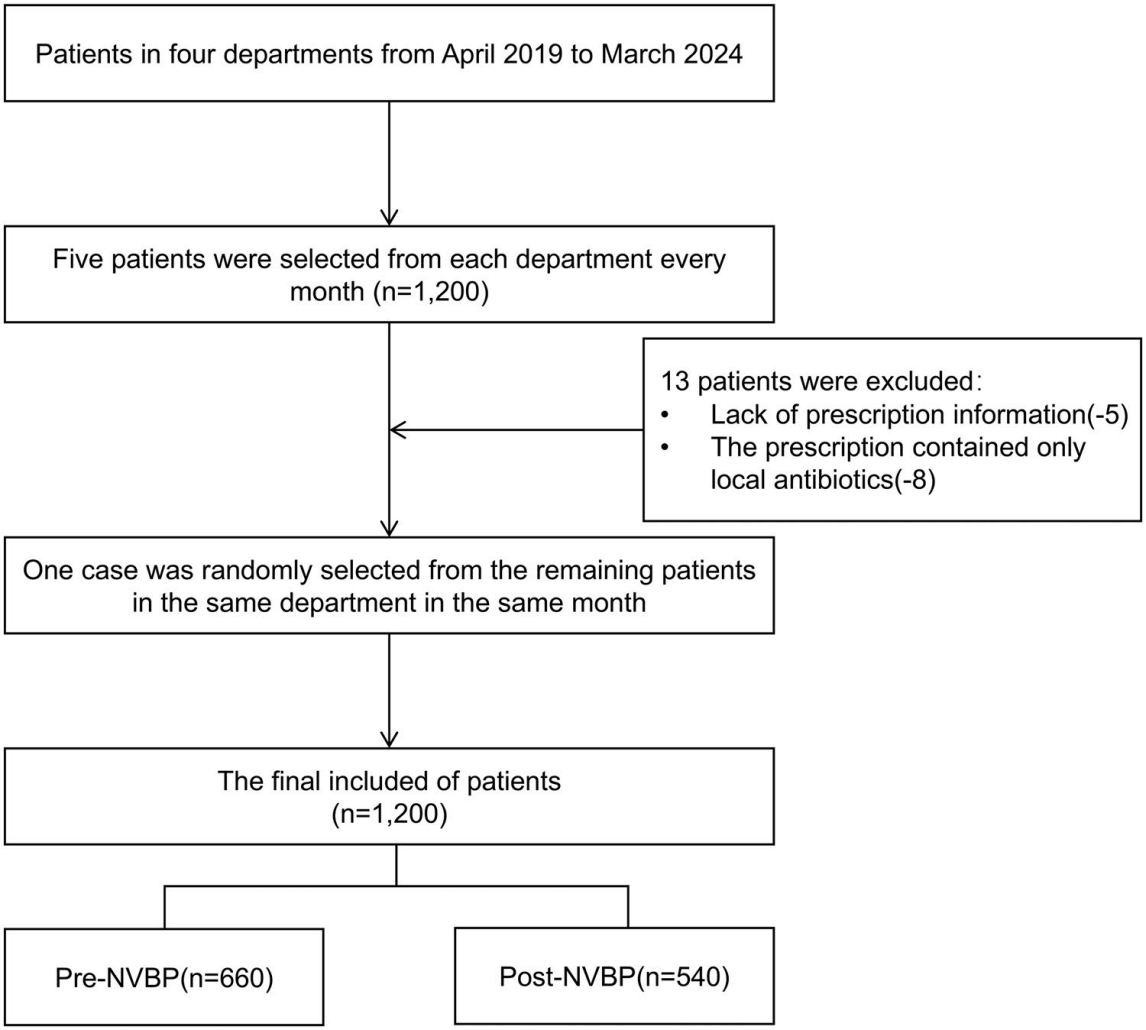
Sample Library Establishment Flowchart.

The data used in this study were sourced from the hospital’s information system. A systematic sampling method was employed, dividing the entire study period by month to ensure a relatively stable monthly data volume. Considering the frequency of antimicrobial drug use, four departments with relatively high usage – respiratory medicine, ICU, orthopaedics, and urology – were chosen as sample source departments. Each month, five inpatient prescription records were randomly selected from each department using a computer-generated random number list applied to all prescriptions for that month. The inclusion criterion was (1) prescriptions issued within the study period at this hospital. Meanwhile, the exclusion criteria were (1) prescriptions for topical antimicrobial preparations, such as external medications, eye drops and mouthwashes; and (2) those with severely missing or invalid information. If a prescription met the exclusion criteria, it was replaced by selecting another prescription from the remaining prescriptions in the same department and month using a computer-generated random number. The study timeframe spanned from April 2019 to March 2024, totalling 5 years, ultimately forming a sample library containing 1200 prescription records, as presented in Figure 1. Data were divided into two groups based on the procurement timeline – pre-NVBP group (April 2019–December 2021) and post-NVBP group (January 2022–March 2024) – to compare changes in relevant indicators before and after NVBP policy implementation.

The extracted data included patient age, sex, diagnosis, admission and discharge dates, surgery name and time, incision type [16] (classified according to the Ministry of Health 2012 Edition of the Guidelines for Completing the First Page of Medical Records: Class 0 for procedures performed *via* natural orifices or percutaneous endoscopy; Class I for clean wounds; Class II for clean-contaminated wounds; and Class III for contaminated wounds), payment method, and healthcare, out-of-pocket, drug, and antimicrobial drug expenditures. The drug data included drug name, specification, dosage form, usage and dosage, frequency, start and stop times, and whether it was an NVBP drug. As of March 2024, the hospital had implemented up to the ninth batch of the national volume-based drug procurement. Supplementary Table 1 presents the antimicrobial drugs included in the procurement.

### 2.3. NVBP policy implementation measures

As presented in Supplementary Figure 1, the management of NVBP drugs was guided by the principle of ‘treating NVBP fulfillment as a political priority, while avoiding one-size-fits-all measures by decomposing targets, cascading goals and monitoring progress monthly.’ This involved the following steps: First, the overall procurement target issued by the medical insurance bureau was decomposed into specific quantity goals for each clinical department based on their historical usage data. These departmental targets were then cascaded to relevant prescribing units. For target quantity determination, the historical actual usage of each department was calculated by generic name. For example, when calculating moxifloxacin consumption, both domestic moxifloxacin and imported Avelox (Bayer) were included, converted, and designated as the department’s target quantity, with particular emphasis on drugs with historically high usage related to prevalent diseases. In the assessment, which included monthly monitoring of progress, the medical insurance bureau evaluated the consumption volume and expenditure share of NVBP-procured drugs relative to the total (both procured and non-procured), requiring this share to exceed 80%; the hospital assessed departmental usage progress of procured drugs monthly. Regarding authority management, strict control was imposed on the use of non-selected drugs, implementing measures such as prescription right restrictions and limited supply while strengthening supervision of similar drugs.

### 2.4. Variable definition and measurement

The primary outcome indicators of this study were antimicrobial drug use volume, use expenditure, and variety structure. The defined daily dose (DDD) method was employed to calculate the frequency of antimicrobial drug use. DDD values were based on the latest data published by the WHO in 2024 [17]. The definitions of key antimicrobial drug categories (e.g. NVBP vs. non-NVBP, unrestricted, restricted, and special-grade agents) and the calculation formulas for all outcome indicators are provided in Supplementary Table 2.

### 2.5. Statistical analysis

This study first quantified the changes in physicians’ antimicrobial prescribing behaviours before and after NVBP policy implementation using descriptive statistics. The Shapiro–Wilk test was employed to determine whether the quantitative variables followed normal distribution. Normally distributed data are expressed as mean ± standard deviation, and group comparisons were made using the independent two-sample *t*-test; non-normally distributed data are expressed as median (interquartile range), and group comparisons were made using the Wilcoxon rank-sum test; finally, categorical variables are expressed as numbers (percentages), and group differences were evaluated using the chi-squared test. To address the issue of multiple comparisons in these pre-post analyses, *P* values were adjusted using the Benjamin–Hochberg false discovery rate (FDR) procedure.

Simultaneously, interrupted time series (ITS) analysis was employed to evaluate both the immediate change and the long-term trend shift of the NVBP policy on the indicators and costs of antimicrobial use. This method is suitable for longitudinal analyses of intervention effects at fixed time points [18]. Taking January 2022 as the intervention point, with time units of one month, we analysed 60 monthly data points (33 pre-intervention, 27 post-intervention). The level and trend changes in outcome variables following policy implementation were estimated using a segmented regression model controlling for baseline trends. The established segmented linear regression model was *Yt = β_0_ + β_1_Timet + β_2_Interventiont + β_3_TimeAfterInterventiont + εt*. The model estimates four key parameters: baseline level (*β_0_*), pre-policy trend (*β_1_*), immediate change after policy (*β_2_*), and change in trend after policy (*β_3_*). The Durbin–Watson test was employed to assess residual autocorrelation. If autocorrelation is present in the linear regression results, the ARIMA model is applied; this model reports the Ljung–Box test result as X-squared. The results were expressed as regression coefficients (*β*), 95% confidence intervals, and *P* values, with *p* < 0.05 considered statistically significant. All statistical analyses were conducted using IBM SPSS 25.0 or GraphPad Prism 9.0. To further test the robustness of the model, sensitivity analysis was conducted by refitting the ITS model after shifting the intervention time point forward and backward by 1 month.

## 3. Results

### 3.1. Baseline characteristics

**Figure 2.**
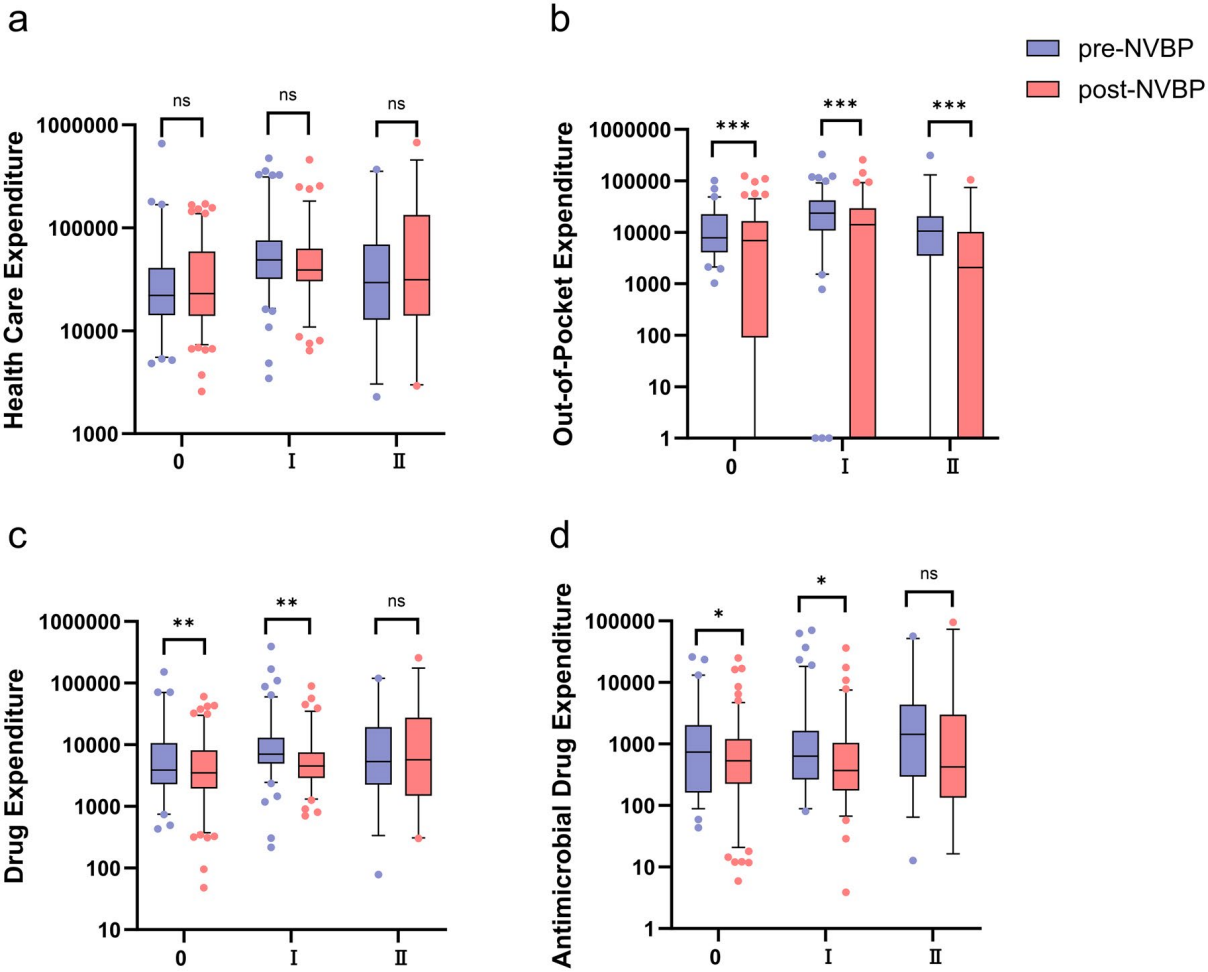
Stratified Comparative Analysis of Cost Indicators for Patients with Class 0, I, and II Incisions. Panel a: Healthcare Expenditure; Panel b: Out-of-Pocket Expenditures; Panel c: Drug Expenditures; Panel d: Antimicrobial Drug Expenditures.

For the period from April 2019 to March 2024, 1200 patients met the relevant inclusion criteria. Their basic characteristics are presented in Table 1. A significant difference was observed in the distribution of incision types between the two groups (*p* < 0.001); thus, this factor may be a potential confounding variable that should be considered and controlled for in subsequent analyses. To determine whether the NVBP policy had a consistent impact across different surgical types, we conducted a stratified analysis of the primary outcome indicators by incision type (see Supplementary Table 3). As illustrated in Figure 2 (a–d), after excluding the interference of incision type differences for patients with Class 0 and Class I incision surgeries, the length of stay, out-of-pocket expenditures, drug expenditures, and antimicrobial drug expenditures following NVBP implementation were significantly lower than those before implementation (all *p* < 0.05). This finding aligns with the overall analysis, indicating that the positive effects of the procurement policy are not driven by changes in the patient mix but instead reflect an independent positive effect. Notably, for patients with Class II incision surgeries, most indicators exhibited no significant improvement, suggesting that the policy effect varies among surgeries of different complexity levels.

**Table 1.** Clinical Characteristics of inpatients before and after NVBP policy implementation.

	Pre-NVBP (*n* = 660)	Post-NVBP (*n* = 540)	*P*
Sex, n (%)			0.239
Male	411 (62.3)	354 (65.6)	
Female	249 (37.7)	186 (34.4)	
Age, M (IQR)	67 (51.0, 76.0)	67 (52.0, 75.0)	0.630
Principal Diagnosis, n (%)			0.415
Malignant tumour and related treatment	152 (23.0)	116 (21.5)	
Trauma and surgical diseases	160 (24.2)	131 (24.3)	
Severe infection and sepsis	53 (8.0)	45 (8.3)	
Cardiocerebrovascular Emergency	54 (8.2)	45 (8.3)	
Acute exacerbation of COPD / asthma	31 (4.7)	14 (2.6)	
Common pneumonia and respiratory tract infection	63 (9.5)	54 (10.0)	
Urinary System Diseases and Related Operations	92 (13.9)	88 (16.3)	
Osteoporosis	14 (2.1)	5 (0.9)	
Digestive system disease	14 (2.1)	15 (2.8)	
Poisoning and metabolic emergencies	8 (1.2)	7 (1.3)	
Nervous system incurable diseases and sequelae	7 (1.1)	3 (0.6)	
Others	12 (1.8)	17 (3.1)	
Comorbidities, n (%)			
Hypertension	267 (40.5)	231 (42.8)	0.416
Diabetes	115 (17.4)	109 (20.2)	0.222
Coronary heart disease and cardiac insufficiency	80 (12.1)	69 (12.8)	0.732
Renal dysfunction	83 (12.6)	68 (12.6)	0.993
History of malignant tumor or current history	43 (6.5)	37 (6.9)	0.816
Incision types, n (%)			**<0.001**
Class 0 incision	148 (22.4)	216 (40.0)	
Class I Incision	174 (26.4)	130 (24.1)	
Class II Incision	40 (6.1)	43 (8.0)	
Class III Incision	3 (0.5)	5 (0.9)	
Medical payment method, n (%)			0.600
Medical Insurance	518 (78.5)	417 (77.2)	
Out-of-pocket	142 (21.5)	123 (22.8)	

Note: Categorical variables are expressed as n (%) and were analysed using the χ^2^ test; age is presented as M (IQR) and was analysed using the Mann–Whitney U-test.

### 3.2. Analysis of antimicrobial drug use

As presented in Table 2, analysis of the antimicrobial drug use structure revealed that the utilization rate of *β*-lactams decreased significantly by 13.85% after FDR adjustment (FDR-adjusted *p* = 0.023). Regarding combination therapy, the overall combination rate exhibited no significant change, but the combination rate of *β*-lactams + fluoroquinolones significantly decreased by 75.93% (FDR-adjusted *p* = 0.023). We also observed numerical trends in other drug categories: decreases in macrolides/lincosamides (−73.33%) and nitroimidazoles (−71.43%), and increases in the proportions of unrestricted agents (+32.00%) and special-grade agents (+31.98%). However, none of these changes remained statistically significant after FDR adjustment (all adjusted *p* > 0.05).

**Table 2.** Changes in antimicrobial drug categories, combination therapy, and usage levels before and after NVBP policy implementation.

	Pre-NVBP (*n* = 427)	Post-NVBP (*n* = 379)	Growth Rate %	*P*	FDR-adjusted *P* value
Antimicrobial Drug Categories					
β-lactams	364 (85.2)	278 (73.4)	−13.85	**<0.001**	**0.023**
Carbapenems	116 (27.2)	108 (28.5)	4.78	0.674	0.861
Fluoroquinolones	111 (26.0)	103 (27.2)	4.62	0.705	0.811
Aminoglycosides	32 (7.5)	24 (6.3)	−16.00	0.517	0.850
Glycopeptides	37 (8.7)	38 (10.0)	14.94	0.507	0.897
Oxazolidinones	18 (4.2)	19 (5.0)	19.05	0.589	0.846
Tetracyclines	29 (6.8)	29 (7.7)	13.24	0.637	0.862
Macrolides/Lincosamides	13 (3.0)	3 (0.8)	−73.33	0.022	0.127
Nitroimidazoles	12 (2.8)	3 (0.8)	−71.43	0.034	0.130
Antifungal drugs	23 (5.4)	22 (5.8)	7.41	0.796	0.832
Combination Therapy	144(33.7)	121 (31.9)	−5.34	0.588	0.902
β-lactam + Aminoglycosides	9 (2.1)	3 (0.8)	−61.90	0.124	0.357
β-lactam + Fluoroquinolones	23 (5.4)	5 (1.3)	−75.93	**0.002**	**0.023**
β-lactam + Nitroimidazole	5 (1.2)	2 (0.5)	−58.33	0.326	0.750
Carbapenems + Aminoglycosides	4 (0.9)	1 (0.3)	−66.67	0.225	0.575
Carbapenems + Fluoroquinolones	29 (6.8)	27 (7.1)	4.41	0.853	0.853
Carbapenems + Glycopeptides	17 (4.0)	20 (5.3)	32.50	0.380	0.728
Carbapenems + Antifungal drug	9 (2.1)	7 (1.8)	−14.29	0.791	0.866
Oxazolidinones + Antifungal drug	2 (0.5)	4 (1.1)	120.00	0.333	0.696
Antimicrobial Drug Usage Levels					
Unrestricted Antimicrobial Drugs	64 (15.0)	75 (19.8)	32.00	0.072	0.237
Restricted Antimicrobial Drugs	398 (93.2)	335 (88.4)	−5.15	0.017	0.130
Special-Grade Antimicrobial Drugs	95 (22.2)	111 (29.3)	31.98	0.022	0.101
Proportion of patients receiving NVBP antimicrobial agents	178 (41.7)	164 (43.3)	3.84	0.649	0.786

Note: Data in the table, the number of patients using antimicrobial drugs (n) and their percentage (%) within the total drug-using patient population for the respective period. Growth rate calculation: growth rate (%) = [(post-NVBP user count − pre-NVBP user count)/pre-NVBP user count] × 100%. Intergroup comparisons were performed using the χ^2^ test or Fisher’s exact test (when the expected frequency is <5). *P* values were adjusted for multiple comparisons using the Benjamini–Hochberg false discovery rate procedure, with an FDR-adjusted *p* < 0.05 considered statistically significant.

As presented in Supplementary Table 4, regarding the intensity of antimicrobial drug use, the median DDDs of antimicrobial agents decreased by 2 units (*p* = 0.002) and that of non-NVBP antimicrobial agents decreased by 1 unit (*p* = 0.005); meanwhile, the median DDDs of NVBP antimicrobial agents remained 0 pre- and post-NVBP policy implementation (*p* = 0.612).

**Figure 3.**
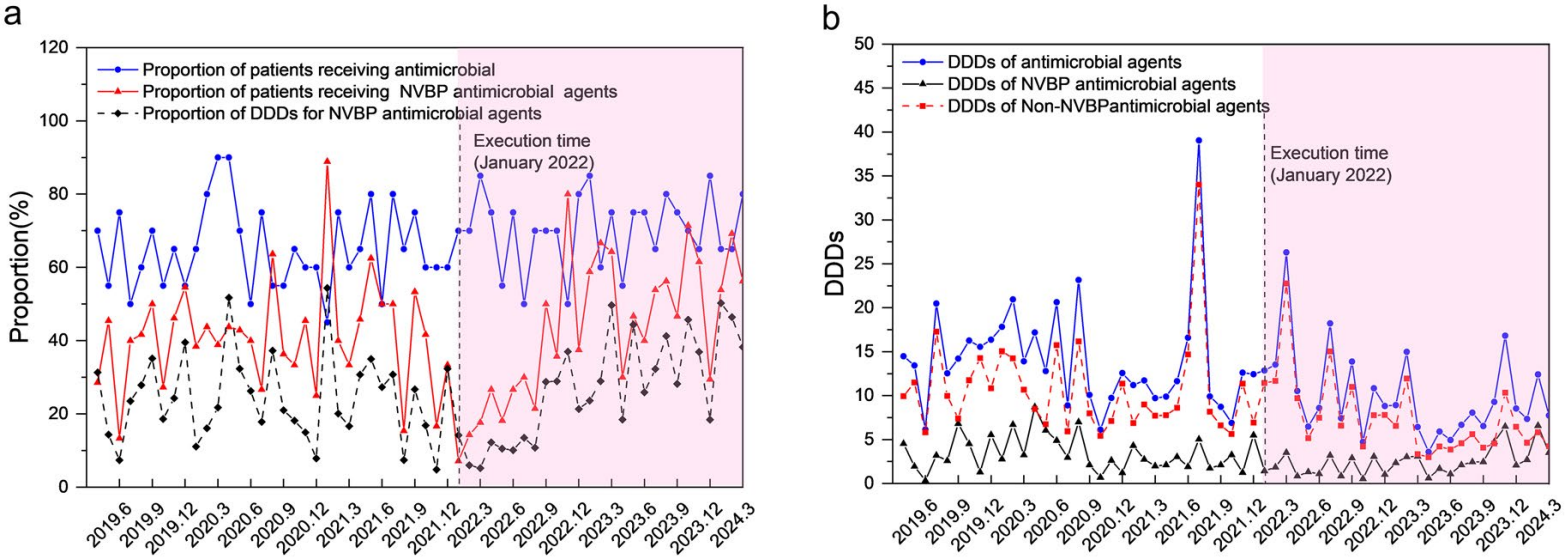
ITS Analysis of Antimicrobial Use Before and After the NVBP Policy Implementation. Panel a: Patient proportions receiving antimicrobial drugs. Panel b: DDDs of antimicrobials.

As presented in Supplementary Table 5, further ITS revealed that post-NVBP policy implementation, the immediate change in the DDDs of NVBP antimicrobial agents did not reach statistical significance (*β_2_* = −1.806 units, *p* = 0.058) but subsequently exhibited a significant upward trend (*β_3_* = 0.135 units/month, *p* = 0.017); the proportion of DDDs for NVBP antimicrobial agents exhibited a significant immediate decrease (*β_2_* = −15.464 units, *p* = 0.009) and a significant upward trend (*β_3_* = 1.400 units/month, *p* < 0.001). Contrarily, the level and trend changes for the DDDs of antimicrobial agents, DDDs of non-NVBP antimicrobial agents, and other indicators exhibited no statistically significant impact (all *p* > 0.05). As presented in Figure 3(a), the proportion of DDDs for NVBP antimicrobial agents continued to increase after a brief initial drop post-NVBP policy implementation, reflecting physicians’ gradually increasing acceptance of NVBP varieties. Figure 3(b) shows that the DDDs of NVBP antimicrobial agents continued to increase, contrasting with non-NVBP antimicrobial agents.

### 3.3. Analysis of antimicrobial drug expenditure and healthcare resource consumption

**Figure 4.**
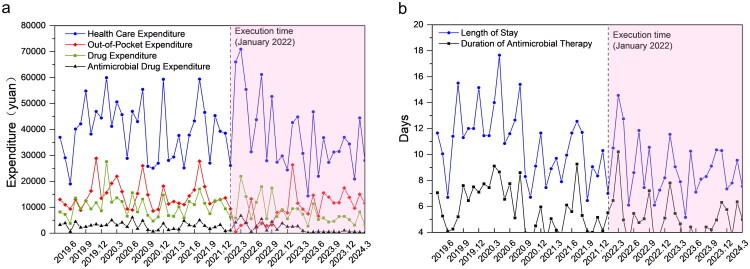
ITS Analysis of Healthcare Expenditures and Utilization Outcomes Before and After NVBP Implementation. Panel a: Trends in healthcare and drug expenditures.; Panel b: Trends in patient length of stay and antimicrobial therapy duration.

Supplementary Table 4 shows that after the implementation of the NVBP policy, multiple cost indicators decreased significantly. Based on the ITS analysis in Supplementary Table 6 and as illustrated in Figure 4(a), antimicrobial drug expenditure showed a short-term increase followed by a sustained decline, while out-of-pocket and overall drug expenditures exhibited clear inflection points of decrease at the time of policy implementation.

In terms of resource consumption, the median length of stay shortened by 1 day compared with the pre-NVBP (*p* < 0.001), and the median duration of antimicrobial therapy decreased by 1 day (*p* = 0.001), as shown in Supplementary Table 4. As presented in Figure 4(b), the monthly data points for both length of stay and duration of antimicrobial therapy appeared lower post-policy, with narrowed fluctuation ranges. However, the ITS analysis (Supplementary Table 6) did not identify a statistically significant change in the level or trend for length of stay (*β_2_* = 0.436 days, *p* = 0.727; *β_3_* = 0.035 days/month, *p* = 0.636).

### 3.4. Sensitivity analysis

As presented in Supplementary Table 7, to test the robustness of the impact of the NVBP policy on antimicrobial use indicators, a sensitivity analysis was conducted by reconstructing the ITS models, shifting the intervention time point forward and backward by 1 month. Results demonstrated that most key indicators – such as out-of-pocket expenditures, antimicrobial drug expenditures, and proportion of DDDs for NVBP antimicrobial agents – maintained significant change trends consistent with the baseline model even after altering the intervention time point. This indicates that the study findings are robust to variations in the intervention time point.

## 4. Discussion

This study systematically evaluated the impact of the NVBP policy on clinical antimicrobial use patterns, medical costs, and resource utilization through a retrospective analysis of antimicrobial drug use data from 1200 inpatients between April 2019 and March 2024 using an ITS model. The results indicate that the NVBP policy is associated with reduced patients’ financial burden, optimized drug use structure, and shorter median hospital stay.

The study’s findings regarding reduced medical costs are consistent with the conclusions of most domestic investigations [19,20]. Specifically, drug prices decreased substantially, as evidenced by significant median reductions in patient out-of-pocket expenditures, drug expenditures, and antimicrobial drug expenditures (*p* < 0.001 for all). Thus, this mechanism effectively alleviates the problem of high medical costs. Notably, antimicrobial drug expenditures showed a brief immediate-level increase post-NVBP policy implementation (*β_2_* = 1476.61yuan, *p* = 0.046), while the proportion of DDDs for NVBP antimicrobial agents exhibited a significant immediate decrease (*β_2_* = −15.464, *p* = 0.009). Following this initial transition period, the positive effects of the policy became apparent: antimicrobial drug expenditures showed a continuous downward trend (*β_3_* = −127.60 yuan/month, *p* = 0.004), and the proportion of DDDs for NVBP antimicrobial agents shifted to a significant monthly upward trend (*β_3_* = 1.400/month, *p* < 0.001), indicating a continuous increase in the proportion of patients receiving NVBP antimicrobials agents and the gradual achievement of the policy’s volume-based goals. Nevertheless, during this transition, it remains crucial to be aware of the risk of NVBP antimicrobial agent overuse, driven by the need to fulfill volume-based targets [21].

From the perspective of prescribing behaviours at the micro level, this study observed that the NVBP policy, through the dual mechanisms of ‘volume guidance’ and ‘price reduction,’ has induced a profound and complex restructuring of antimicrobial drug utilization patterns. The utilization rate of *β*-lactams, the fundamental agents accounting for the majority of prescriptions, decreased significantly by 13.85%. Contrary to expectations of reduced high-end drug use, a notable numerical increase in the proportion of special-grade antimicrobial agents was observed (+31.98%, from 22.2% to 29.3%), with the utilization rate of carbapenems also increasing by 4.78%. Both increases did not reach statistical significance after adjustment for multiple comparisons. Nevertheless, as carbapenems are crucial ‘last-line’ drugs for treating resistant Gram-negative bacterial infections, any upward trend in their utilization requires considerable attention [22]. Coupled with the severe situation of increasing carbapenem-resistant Gram-negative bacilli detection rates continuously reported by China’s CHINET surveillance network if the trend observed in this study is validated and widespread, it could potentially exacerbate bacterial resistance and pose a serious threat to public health [9,14].

This phenomenon is possibly associated with the following mechanisms. (1) Concerns regarding ‘therapeutic downgrading’: Clinicians may doubt the efficacy of non-special-grade category drugs selected in the NVBP, such as certain *β*-lactams. Driven by risk aversion, they might directly choose higher-level, special-grade antimicrobials [23]. (2) Changes in case mix: Although baseline characteristics exhibited minimal intergroup differences, unavailable clinical data – such as infection severity – might indicate that more patients with severe infections were admitted post-NVBP policy implementation, naturally requiring more high-level antibiotics. (3) Gaps in policy implementation: Hospitals may inadequately enforce core control measures for special-grade antimicrobials, such as prescription authorization approvals and requirements for microbiological testing, resulting in insufficient restrictions on their use. This markedly demonstrates the complexity of clinical prescribing behaviours: for common infections, physicians actively respond to the policy by preferentially selecting fundamental NVBP drugs. However, for severe and complex infections, clinical need remains the absolute dominant factor in prescribing decisions, rendering the policy’s economic leverage ineffective in such micro-level clinical scenarios.

As regards combination therapy, the substantial reduction in non-standard regimens, such as s *β*-lactams + fluoroquinolones (−75.93%, *p* = 0.023), may be attributed to the NVBP policy’s impact on prescribing incentives: as the prices of certain fluoroquinolones (e.g. moxifloxacin) dropped sharply after being included in the procurement catalogue, their previous economic advantage in broad-spectrum combination regimens diminished. Concurrently, hospitals intensified scrutiny over non-guideline-based combinations to meet antimicrobial stewardship goals aligned with the policy’s efficiency drive. This stands in sharp contrast to the stable or increased utilization rates of precise combination regimens that target multidrug-resistant infections, such as carbapenems + glycopeptides. This suggests that the NVBP policy has, at the micro level, purified the prescribing environment by reducing certain prescription behaviours driven by nonclinical motivations.

This study also identified a noteworthy ‘cost paradox’: while antimicrobial drug expenditures and drug expenditure significantly decreased, healthcare expenditure showed no considerable change. This phenomenon can be explained from a micro-behavioural perspective: the reduction in drug expenditures may have freed up other spending avenues. For example, physicians may have increased diagnostic tests and laboratory examinations or utilized non-procured high-cost medical consumables to compensate for hospital revenue loss or alleviate clinical risks. Concurrently, the rigid demand for special-grade, high-end antimicrobials is accompanied by high comprehensive treatment costs. This finding serves as a warning that sole reliance on drug NVBP is insufficient for controlling total healthcare expenditures. It is necessary to synergistically advance payment system reforms at the micro level, such as those represented by diagnosis-related groups [24].

This study has certain limitations. First, as a single-centre retrospective study, the generalizability of its findings may be limited. Second, as key clinical data such as patient infection severity, microbiological test results, and antimicrobial susceptibility profiles could not be obtained, the clinical drivers of the observed changes in prescribing behaviours could not be analysed in depth. Finally, although ITS analysis suggests associations, it does not fully exclude the potential confounding effects of other concurrent policies or events, such as the COVID-19 pandemic or changes in hospital infection control measures.

## 5. Conclusion

Using ITS analysis, this study demonstrates that while the NVBP policy significantly reduced patient antimicrobial expenditures and financial burden, it was accompanied by trends warranting attention, such as an increased proportion of special-grade antimicrobials and rising carbapenem use. Although cost-containment goals were met, these shifts may pose challenges to rational drug use and antimicrobial resistance control. Therefore, future policies should more closely integrate with hierarchical antimicrobial stewardship, strengthen real-time monitoring and evaluation of high‑level antimicrobial use, and promote multicentre longitudinal research to systematically assess the policy’s long-term impact on resistance and clinical outcomes, thereby providing a robust evidence base for ongoing policy refinement.

## Supplementary Material

Supplementary Material.docx

## Data Availability

As data contain personal health information of individuals, and data were strictly used under license for the current study. The datasets used and/or analysed during the current study are available from the corresponding author on reasonable request.

## Declaration of generative Al and Al-assisted technologies in the writing process

During the preparation of this work, the authors utilized DeepSeek for assistance in refining the text’s readability and overall flow. Following this, the authors thoroughly reviewed and revised the content as necessary and assume full responsibility for the publication’s content.
