# Supplementary material for: Impact of national volume-based procurement on physicians’ antimicrobial prescribing behaviours: an interrupted time series analysis of 1200 prescriptions in a tertiary hospital
Source: Ann Med. 2026 Feb 8;58(1):2624209. doi: 10.1080/07853890.2026.2624209 (PMC12888359; doi:10.1080/07853890.2026.2624209)
Supplement: Supplementary Material.docx [file IANN_A_2624209_SM9739.docx]

**Supplementary Table 1 Antimicrobial Drugs Included in the Volume-Based Procurement.**

| Antimicrobial Agents | Brand Name | Specification |
| --- | --- | --- |
| Linezolid and Glucose Injection | Hengjie 0.2g | 0.2g/100ml |
|  | Tianli | 0.2g/100ml |
|  | Hengjie 0.6g | 0.6g/300ml |
|  | Zyvox | 0.6g/300ml |
| Cefuroxime Sodium for Injection | Runze | 0.75g |
|  | Zinacef | 0.75g |
| Ceftriaxone Sodium for Injection | Yingpaiqi | 1g |
|  | Rocephin | 1g |
| Cefazolin Sodium for Injection | Xintaitong | 1g |
| Ceftazidime for Injection | Yingbeigi | 1g |
|  | Lingxun | 0.5g |
|  | Anduilin | 0.75g |
| Azithromycin for Injection | Ruiqi | 0.5g |
|  | Zithromax | 0.5g |
|  | Qixian | 0.125g |
|  | Qifa | 0.25g |
| Cefazolin Sodium Pentahydrate for Injection | Xintailin | 1g |
| Fluconazole and Sodium Chloride Injection | Yilikang | 0.2g/100ml |
| Ceftizoxime Sodium for Injection | Epocelin | 1g |
| Cefoperazone Sodium and Sulbactam Sodium for Injection | Sulperazon | 1.5g (1g+0.5g) |
| Cefoperazone Sodium and Sulbactam Sodium (2:1) for Injection | Luobei | 1.5g (1.0g/0.5g) |
| Cefotiam Hydrochloride for Injection | Fengtixin | 0.5g, 0.25g |
|  | Haisco | 1.0g |
|  | Peiluoxin | 1g |
| Moxifloxacin Hydrochloride and Sodium Chloride Injection | Moxile | 0.4g/250ml |
|  | Anruotai | 0.4g/250ml |
|  | Avelox | 0.4g/250ml |
| Moxifloxacin Injection | Baimeinuo | 0.4g/20ml |

**Supplementary Table 2 Definitions of Antimicrobial Drug Categories and Key Outcome Indicators.**

| Category / Indicator | Definition / Calculation Formula |
| --- | --- |
| Category |  |
| NVBP drugs | Antimicrobial agents included in the national volume-based procurement catalog as of the study period |
| Non-NVBP drugs | Antimicrobial agents not included in the aforementioned procurement catalog |
| Unrestricted antimicrobial drugs | Agents that can be prescribed routinely by licensed physicians without special restrictions |
| Restricted antimicrobial drugs | Agents whose use requires approval from a senior physician or an antimicrobial stewardship team, typically due to broader spectrum, higher cost, or greater potential for driving resistance |
| Special-grade antimicrobial drugs | A subset of restricted agents, reserved for the treatment of life-threatening infections caused by multidrug-resistant organisms, as per China’s Administrative Measures for the Clinical Application of Antimicrobial Drugs. Their use is subject to the strictest level of authorization and monitoring |
| Key Outcome Indicators |  |
| Defined Daily Doses (DDDs) | Total antimicrobial drug consumption (g) / DDD |
| Duration of Antimicrobial Therapy | Actual number of days of antimicrobial drug use |
| Proportion of DDDs for NVBP Antimicrobial Agents | (DDDs of NVBP antimicrobial agents / DDDs of antimicrobial agents) × 100% |
| Proportion of an Antimicrobial Drug Category | (Number of patients using a specific category of antimicrobial drugs / Total number of patients using antimicrobial drugs) × 100% |
| Proportion of Combination Therapy | (Number of patients receiving combination antimicrobial therapy / Total number of patients using antimicrobial drugs) × 100% |
| Proportion of a Specific Drug Combination | (Number of patients receiving a specific drug combination / Total number of patients using antimicrobial drugs) × 100% |
| Proportion of Patients Receiving NVBP Antimicrobial Drugs | (Number of patients receiving NVBP drugs / Total number of patients using antimicrobial drugs) × 100% |

**Supplementary Table 3 Stratified Analysis by Incision Category.**

| Incision type |  | Pre - NVBP (n=427) | Post - NVBP (n=379) | Median Difference | *P* |
| --- | --- | --- | --- | --- | --- |
| Class 0 Incision |  |  |  |  |  |
|  | Length of Stay (days) | 8 (4, 13) | 6.50 (2, 10.75) | -1.50 | **0.008** |
|  | Health Care Expenditure (yuan) | 20451.52 (10766.72, 41788.54) | 17256.41 (8388.03, 39955.29) | -3195.11 | 0.131 |
|  | Out-of-Pocket Expenditure (yuan) | 6619.90 (3138.15, 18033.30) | 2927.70 (0, 10966.74) | -3692.20 | **<0.001** |
|  | Drug Expenditure (yuan) | 4149.78 (1833.17, 10655.39) | 2976.15 (1217.89, 6388.32) | -1173.63 | **0.005** |
|  | Antimicrobial Drug Expenditure (yuan) | 662.18 (88.60, 2556.75) | 402.87 (12.05, 1298.72) | -259.23 | **0.022** |
| Class I Incision |  |  |  |  |  |
|  | Length of Stay (days) | 10 (7, 17.25) | 9 (6, 14) | -1.00 | **0.008** |
|  | Health Care Expenditure (yuan) | 31855.67 (16673.75, 55568.87) | 33199.55 (18502.67, 51768.48) | 1343.88 | 0.131 |
|  | Out-of-Pocket Expenditure (yuan) | 13115.01 (7969.72, 28785.19) | 9811.09 (0, 21855.65) | -3303.93 | **<0.001** |
|  | Drug Expenditure (yuan) | 5355.01 (2484.23, 8863.52) | 3315.02 (2016.36, 6364.03) | -2039.99 | **0.005** |
|  | Antimicrobial Drug Expenditure (yuan) | 287.45 (0, 1226.5) | 208.11 (0, 722.78) | -79.34 | **0.022** |
| Class II Incision |  |  |  |  |  |
|  | Length of Stay (days) | 10 (7, 22.25) | 7 (3, 18) | -3.00 | 0.343 |
|  | Health Care Expenditure (yuan) | 27043.29 (9718.23, 63197.43) | 20891.68 (8669.03, 104165.84) | -6151.61 | 0.985 |
|  | Out-of-Pocket Expenditure (yuan) | 9784.91 (3151.58, 20357.51) | 1601.82 (0, 8841.32) | -8183.09 | **<0.001** |
|  | Drug Expenditure (yuan) | 4549.89 (1619.59, 13760.94) | 2986.09 (588.64, 16313.83) | -1563.80 | 0.572 |
|  | Antimicrobial Drug Expenditure (yuan) | 963.71 (88.6, 3336.72) | 354.40 (31.4, 2692.36) | -609.31 | 0.230 |

Note: Data are expressed as median (interquartile range). Intergroup comparisons were performed using the Mann–Whitney U test. DDD: defined daily dose; DDDs: defined daily doses;

**Supplementary Table 4 Changes in Antimicrobial Drug Use Intensity and Medical Costs Before and After NVBP Policy Implementation.**

|  | Pre-NVBP (n=427) | Post-NVBP (n=379) | Median Difference | *P* |
| --- | --- | --- | --- | --- |
| Length of Stay (days) | 10 (6, 17) | 9 (5, 13) | -1 | **<0.001** |
| Health Care Expenditure (yuan) | 28335.82 (13044.01, 57905.94) | 27196.98 (13289.02, 53275.04) | -1138.84 | 0.564 |
| Out-of-Pocket Expenditure (yuan) | 9622.43 (3456.93, 25313.12) | 3781.5 (0, 15588.51) | -5840.93 | **<0.001** |
| Drug Expenditure (yuan) | 5873.7 (2741.14, 13076.1) | 3949.8 (2092.08, 11009.17) | -1923.9 | **<0.001** |
| Antimicrobial Drug Expenditure (yuan) | 1222.29 (412.1, 3521.53) | 592.6 (211.08, 1665.46) | -629.69 | **<0.001** |
| Duration of antimicrobial therapy (days) | 7 (3, 11) | 6 (3, 9) | -1 | **0.001** |
| DDDs of antimicrobial agents (DDD) | 7.5 (3, 16.79) | 5.5 (2.67, 11.5) | -2 | **0.002** |
| DDDs of NVBP antimicrobial agents (DDD) | 0 (0, 5) | 0 (0, 3.25) | 0 | 0.612 |
| DDDs of Non-NVBP antimicrobial agents (DDD) | 5 (1.3, 12) | 4 (1, 9) | -1 | **0.005** |
| Proportion of DDDs for NVBP antimicrobial agents (Proportion) | 0 (0, 0.46) | 0 (0, 0.51) | 0 | 0.376 |

Note: Data are expressed as median (interquartile range). Intergroup comparisons were performed using the Mann–Whitney U test. DDD: defined daily dose; DDDs: defined daily doses.

**Supplementary Table 5 ITS Analysis of Changes in Antimicrobial Drug Use Intensity and Costs After NVBP Policy Implementation**

| Category |  | Estimate | 95%CI | *P* |
| --- | --- | --- | --- | --- |
| Proportion of patients receiving antimicrobial |  |  |  |  |
|  | *β*_2_ | 3.439 | (-7.99, 14.87) | 0.549 |
|  | *β*_3_ | 0.171 | (-0.50, 0.84) | 0.612 |
|  | DW | 2.212 |  | 0.698 |
| Duration of antimicrobial therapy |  |  |  |  |
|  | *β*_2_ | 0.403 | (-1.21, 3.01) | 0.403 |
|  | *β*_3_ | 0.739 | (-0.10, 0.14) | 0.739 |
|  | DW | 7.807 |  | 0.648 |
| DDDs |  |  |  |  |
|  | *β*_2_ | 0.088 | (-5.68, 5.85) | 0.976 |
|  | *β*_3_ | -0.144 | (-0.48, 0.20) | 0.410 |
|  | DW | 2.012 |  | 0.710 |
| Proportion of patients receiving NVBP antimicrobial agents |  |  |  |  |
|  | *β*_2_ | -22.771 | (-38.42, -7.12) | **0.005** |
|  | *β*_3_ | 1.555 | (0.63, 2.48) | **0.001** |
|  | DW | 2.169 |  | 0.840 |
| DDDs of NVBP antimicrobial agents |  |  |  |  |
|  | *β*_2_ | -1.806 | (-3.64, 0.02) | 0.058 |
|  | *β*_3_ | 0.135 | (0.02, 0.24) | **0.017** |
|  | DW | 2.024 |  | 0.770 |
| DDDs of Non-NVBP antimicrobial agents |  |  |  |  |
|  | *β*_2_ | 1.684 | (-3.20, 6.57) | 0.502 |
|  | *β*_3_ | -0.288 | (-0.58, 0.00) | 0.054 |
|  | DW | 1.993 |  | 0.616 |
| Proportion of DDDs for NVBP antimicrobial agents |  |  |  |  |
|  | *β*_2_ | -15.464 | (-26.86, -4.07) | **0.009** |
|  | *β*_3_ | 1.400 | (0.73, 2.07) | **<0.001** |
|  | DW | 2.266 |  | 0.552 |

Note: *Estimate,* the coefficient value, with positive values indicating an increase and negative values indicating a decrease; CI, confidence interval; DDDs, defined daily doses.

**Supplementary Table 6 ITS Analysis of Changes in Medical Costs After NVBP Policy Implementation**

| Category |  | Estimate | 95%CI | *P* |
| --- | --- | --- | --- | --- |
| Health Care Expenditure |  |  |  |  |
|  | *β*_2_ | 9191.288 | (-3095.60, 21478.17) | 0.148 |
|  | *β*_3_ | -797.053 | (-1520.43, -73.68) | **0.035** |
|  | DW | 2.065 |  | 0.812 |
| Out-of-Pocket Expenditure |  |  |  |  |
|  | *β*_2_ | -6030.892 | (-12556.18, 494.40) | 0.07 |
|  | *β*_3_ | 490.93 | (69.40, 912.46) | **0.022** |
|  | X-squared | 9.100 |  | 0.523 |
| Drug Expenditure |  |  |  |  |
|  | *β*_2_ | 1793.243 | (-2799.63, 6386.12) | 0.437 |
|  | *β*_3_ | -315.455 | (-585.85, -45.05) | **0.023** |
|  | DW | 2.264 |  | 0.566 |
| Antimicrobial Drug Expenditure |  |  |  |  |
|  | *β*_2_ | 1476.605 | (27.48, 2925.73) | **0.046** |
|  | *β*_3_ | -127.603 | (-212.92, -42.29) | **0.004** |
|  | DW | 2.233 |  | 0.624 |
| Length of Stay |  |  |  |  |
|  | *β*_2_ | 0.436 | (-2.06, 2.93) | 0.727 |
|  | *β*_3_ | 0.035 | (-0.11, 0.18) | 0.636 |
|  | DW | 1.713 |  | 0.112 |

Note: Estimate, the coefficient value, with positive values indicating an increase and negative values indicating a decrease; CI, confidence interval.

**Supplementary Table 7 Sensitivity Analysis Results.**

| Category | Term | Base Model | | Intervention-advanced Model | | Intervention-delayed Model |  |
| --- | --- | --- | --- | --- | --- | --- | --- |
|  |  | Estimate (95%CI) | *P* | Estimate (95%CI) | *P* | Estimate (95%CI) | *P* |
| Length of Stay | *β_2_* | 0.436 (-2.06, 2.93) | 0.727 | 0.593 (-1.89, 3.07) | 0.634 | 1.170 (-1.32, 3.66) | 0.351 |
|  | *β_3_* | 0.035 (-0.11, 0.18) | 0.636 | 0.037 (-0.11, 0.18) | 0.611 | 0.021 (-0.13, 0.17) | 0.778 |
|  | *DW* | 1.71 | 0.063 | 1.70 | 0.057 | 1.70 | 0.058 |
| Health Care Expenditure | *β_2_* | 9191.288 (-3366.66, 21749.24) | 0.148 | 8381.362 (-4189.67, 20952.40) | 0.187 | 13489.927 (1174.10, 25805.76) | **0.032** |
|  | *β_3_* | -797.053 (-1536.39, -57.72) | **0.035** | -727.666 (-1458.89, 3.56) | 0.051 | -931.164 (-1668.95, -193.38) | **0.014** |
|  | *DW* | 2.07 | 0.441 | 2.01 | 0.364 | 2.02 | 0.368 |
| Out-of-Pocket Expenditure | *β_2_* | -10960.802 (-16301.75, -5619.85) | **<0.001** | -10159.166 (-15613.03, -4705.30) | **<0.001** | -10747.968 (-16119.48, -5376.46) | **<0.001** |
|  | *β_3_* | 380.979 (66.54, 695.42) | **0.018** | 301.906 (-15.33, 619.14) | 0.062 | 454.979 (133.20, 776.76) | **0.006** |
|  | *DW* | 1.73 | 0.072 | 1.70 | 0.057 | 1.66 | 0.040 |
| Drug Expenditure | *β_2_* | 1793.243 (-2799.63, 6386.12) | 0.437 | 1517.190 (-3068.21, 6102.59) | 0.510 | 2957.533 (-1610.93, 7526.00) | 0.200 |
|  | *β_3_* | -315.455 (-585.86, -45.06) | **0.023** | -300.526 (-567.25, -33.80) | **0.028** | -347.918 (-621.60, -74.24) | **0.014** |
|  | *DW* | 2.26 | 0.737 | 2.25 | 0.715 | 2.31 | 0.795 |
| Antimicrobial Drug Expenditure | *β_2_* | 1476.605 (27.48, 2925.73) | **0.046** | 957.722 (-520.22, 2435.67) | 0.200 | 1940.633 (530.40, 3350.86) | **0.008** |
|  | *β_3_* | -127.603 (-212.92, -42.29) | **0.004** | -114.512 (-200.48, -28.54) | **0.010** | -145.703 (-230.18, -61.22) | **0.001** |
|  | *DW* | 2.23 | 0.694 | 2.16 | 0.586 | 2.29 | 0.767 |
| Proportion of Patients Receiving Antimicrobial | *β_2_* | 3.440 (-7.99, 14.9) | 0.549 | 1.530 (-9.87, 12.9) | 0.789 | 2.720 (-8.79, 14.2) | 0.638 |
|  | *β_3_* | 0.171 (-0.50, 0.84) | 0.612 | 0.203 (-0.46, 0.87) | 0.542 | 0.160 (-0.53, 0.85) | 0.643 |
|  | *DW* | 2.21 | 0.665 | 2.21 | 0.662 | 2.21 | 0.669 |
| Duration of Antimicrobial Therapy | *β_2_* | 0.845 (-0.93, 2.62) | 0.345 | 0.503 (-1.28, 2.28) | 0.574 | 0.810 (-0.98, 2.60) | 0.369 |
|  | *β_3_* | 0.009 (-0.10, 0.11) | 0.865 | 0.016 (-0.09, 0.12) | 0.755 | 0.004 (-0.10, 0.11) | 0.944 |
|  | *DW* | 1.48 | **0.006** | 1.48 | **0.006** | 1.48 | **0.006** |
| DDDs | *β_2_* | 0.089 (-5.81, 5.98) | 0.976 | 0.089 (-5.78, 5.96) | 0.976 | -0.066 (-6.00, 5.86) | 0.982 |
|  | *β_3_* | -0.144 (-0.49, 0.20) | 0.410 | -0.142 (-0.48, 0.20) | 0.407 | -0.143 (-0.50, 0.21) | 0.423 |
|  | *DW* | 2.01 | 0.360 | 2.01 | 0.360 | 2.01 | 0.360 |
| Proportion of Patients Receiving NVBP Antimicrbial Agents | *β_2_* | -0.228 (-0.38, -0.071) | **0.005** | -0.236 (-0.39, -0.08) | **0.004** | -0.148 (-0.31, 0.01) | 0.073 |
|  | *β_3_* | 0.016 (0.01, 0.02) | **0.013** | 0.014 (0.01, 0.02) | **0.003** | 0.016 (0.01, 0.03) | **0.002** |
|  | *DW* | 2.17 | 0.601 | 2.32 | 0.800 | 2.10 | 0.499 |
| DDDs of NVBP Antimicrobial Agents | *β_2_* | -1.810 (-3.68, 0.06) | 0.058 | -0.986 (-2.90, 0.92) | 0.305 | -1.550 (-3.44, 0.341) | 0.106 |
|  | *β_3_* | 0.135 (0.02, 0.25) | **0.017** | 0.118 (0.01, 0.23) | **0.038** | 0.144 (0.03, 0.26) | **0.013** |
|  | *DW* | 2.02 | 0.379 | 2.03 | 0.389 | 2.08 | 0.468 |
| DDDs of Non-NVBP Antimicrobial Agents | *β_2_* | 1.680 (-3.31, 6.68) | 0.502 | 0.847 (-4.14, 5.83) | 0.735 | 1.290 (-3.73, 6.31) | 0.609 |
|  | *β_3_* | -0.288 (-0.58, 0.01) | 0.055 | -0.271 (-0.56, 0.02) | 0.066 | -0.296 (-0.60, 0.01) | 0.054 |
|  | *DW* | 1.99 | 0.333 | 1.99 | 0.333 | 2.00 | 0.342 |
| Proportion of DDDs for NVBP Antimicrobial Agents | *β_2_* | -0.155 (-0.27, -0.04) | **0.009** | -0.123 (-0.24, -0.01) | **0.0398** | -0.138 (-0.25, -0.02) | **0.020** |
|  | *β_3_* | 0.014 (0.01, 0.02) | **<0.001** | 0.013 (0.01, 0.02) | **<0.001** | 0.015 (0.01, 0.02) | **<0.001** |
|  | *DW* | 2.27 | 0.739 | 2.28 | 0.761 | 2.28 | 0.762 |

Note: Estimate, the coefficient value, with positive values indicating an increase and negative values indicating a decrease; CI, confidence interval; DDDs, defined daily doses.


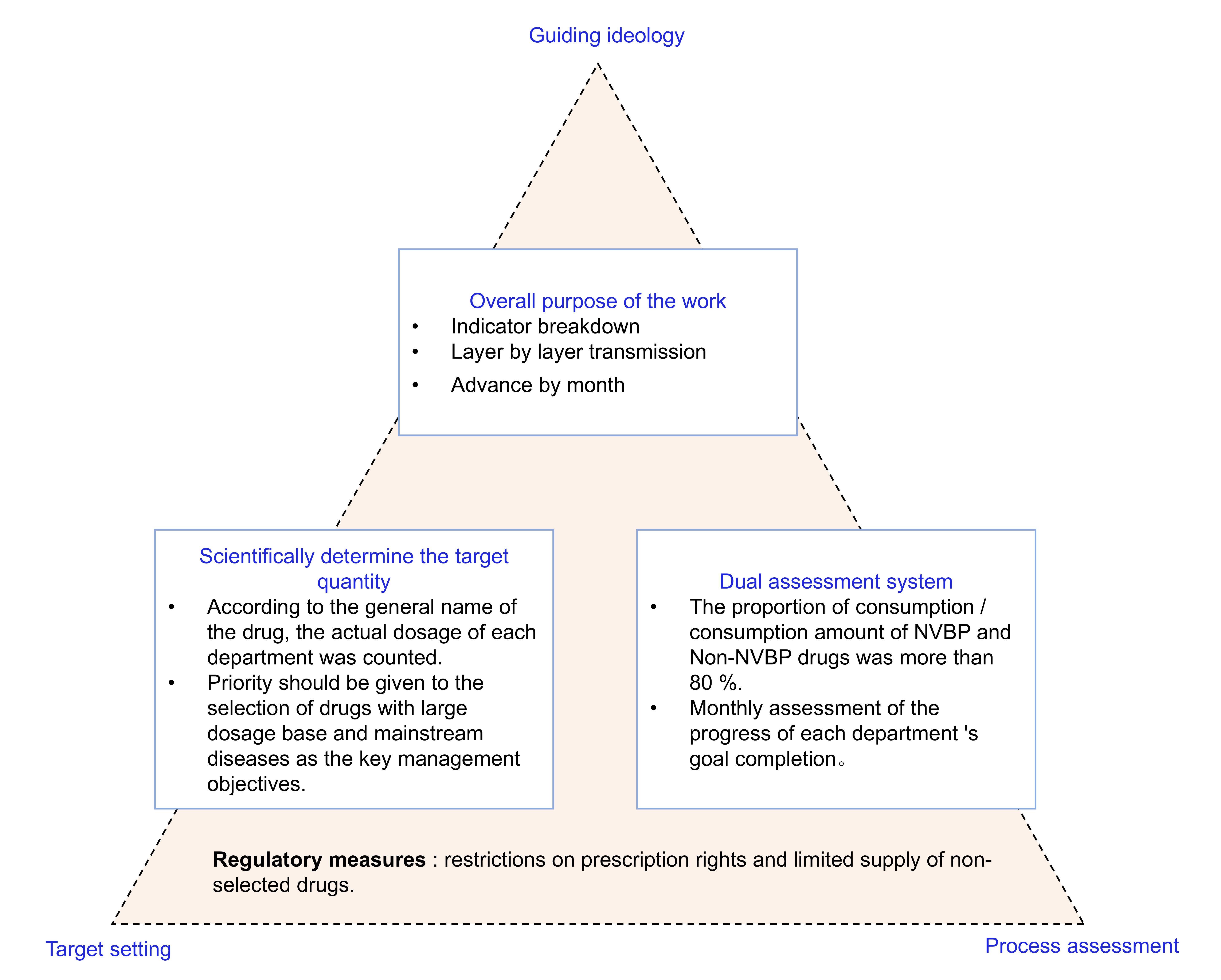


**Supplementary Figure 1: NVBP Policy Implementation Status**
